# Short-term and Long-term Stability of the Autism Diagnostic Observation Schedule (ADOS-2) Calibrated Comparison Scores (CCS) and Classification Scores in Youth with Down Syndrome or Fragile X Syndrome with Intellectual Disability

**DOI:** 10.1007/s10803-024-06535-8

**Published:** 2024-09-09

**Authors:** Angela John Thurman, Amanda Dimachkie Nunnally, Vivian Nguyen, Elizabeth Berry-Kravis, Audra Sterling, Jamie Edgin, Debra Hamilton, Jeannie Aschkenasy, Leonard Abbeduto

**Affiliations:** 1 MIND Institute, University of California, Davis, 2825 50th Street, Room 2335, Sacramento, CA 95817 USA; 2 Department of Psychiatry and Behavioral Sciences, University of California Davis Medical Center, Sacramento, USA; 3 Departments of Pediatrics, Neurological Sciences and Anatomy and Cell Biology, Rush University Medical Center, Chicago, USA; 4 Waisman Center and Department of Communication Sciences and Disorders, University of Wisconsin-Madison, Madison, USA; 5 Department of Psychology, Virginia Tech, Blacksburg, USA; 6 Department of Human Genetics, Emory University, Atlanta, USA; 7 Department of Pediatrics, Rush University Medical Center, Chicago, USA

**Keywords:** Autism symptomatology, ASD classification, Down syndrome, Fragile X syndrome, Intellectual disability

## Abstract

Autism diagnosis in individuals with fragile X syndrome (FXS) or Down syndrome (DS) with co-occurring intellectual disability is complex since clinicians often must consider other co-occurring behavioral features. Understanding how best to assess the features of autism in individuals with these conditions is crucial. In this study, we consider the short-term and long-term psychometric consistency of the Autism Diagnostic Observation Schedule-2 (ADOS-2) calibrated comparison scores (CCSs) and ASD classifications in individuals with FXS or DS. 76 individuals with DS (39 males; *M*_*age*_ = 15.27) and 90 individuals with FXS (71 males; *M*_*age*_ = 14.52 years) completed an assessment battery (ADOS-2, abbreviated IQ assessment and semi-structured language sample) at three timepoints (initial visit, short-term stability visit, long-term stability visit). All CCSs were found to have short-and long-term consistency for both groups, with lowest reliability scores for the repetitive behaviors (RRB) CCSs. Decreased reliability of RRB CCSs was found in the DS group than the FXS group. Variable short- and long-term ASD classifications were observed in both groups, with significantly higher variability in the DS group. Across groups, participants with variable classifications had lower ADOS-2 CCSs and higher language scores than those with stable ASD classifications. In the FXS group, those with variable classifications earned higher cognitive scores than did participants with stable ASD classifications. These findings highlight the high incidence of autism symptomatology in individuals with DS or FXS and co-occurring intellectual disability, while elucidating the short- and long-term variability of symptom expression in the context of structured observational tasks such as the ADOS-2.

## Introduction

Autism spectrum disorder (ASD) is a behaviorally defined condition characterized by two core features: social communication challenges and the presence of repetitive and stereotyped behaviors (American Psychiatric, 2013). Importantly, autism is heterogeneous in terms of etiology. In the general population, the occurrence of autism is approximately 1 in 36 children (Maenner et al., 2023). In 10% of cases, autism is observed in individuals with a known genetic syndrome (Abrahams & Geschwind, 2008). Compared to rates observed in the general population, the likelihood of presenting with autism symptomatology is higher for individuals with several neurogenetic conditions, especially those associated with intellectual disability. For example, with an estimated co-occurrence rate of 60%, fragile X syndrome (FXS) is the leading single-gene cause of autism (Clifford et al., 2007; Harris et al., 2008; Kaufmann et al., 2017; Klusek et al., 2014; Philofsky et al., 2004), as well as the leading inherited cause of intellectual disability. Similarly, 16 to 42% of individuals with Down syndrome (DS), which is the leading genetic cause of intellectual disability, are diagnosed as having a co-occurring ASD diagnosis (DiGuiseppi et al., 2010; Dimachkie Nunnally et al., 2021; Oxelgren et al., 2017; Warner et al., 2017).

Despite evidence that some neurogenetic conditions are associated with a higher occurrence of autism symptomatology, much remains to be understood. Detailed investigations of the presence of autism symptomatology in neurogenetic intellectual disability conditions are critical for understanding the clinical implications of these symptoms for affected individuals. The assessment of autism in individuals with neurogenetic conditions is complex because those conducting the evaluation have to consider the potential influences of the co-occurring presence of intellectual disability, language delays, or other clinical features (e.g., attention and anxiety). Nonetheless, the accurate identification of autism in individuals with neurogenetic conditions is crucial for providing appropriate care, supporting optimal outcomes, preventing misdiagnosis, and advancing both research and clinical practice (e.g., Klusek et al., 2014; Spinazzi et al., 2023a, 2023b). A crucial first step in addressing the complex issues of co-occurrence, however, is understanding how best to assess the features of autism in individuals with these neurogenetic conditions.

The Autism Diagnostic Observation Schedule-2 (Lord et al., 2012) is a gold-standard autism assessment tool widely used in research and clinical settings to characterize the presence of autism symptomatology (Gotham et al., 2007; Maddox et al., 2020; Risi et al., 2006). Indeed, using a standardized observational context to form behavioral observations can be valuable for framing clinical impressions of an individual’s skills and behavioral repertoire. However, given the potential influences of other co-occurring behavioral features (e.g., intellectual disability), the ADOS-2 scores were not explicitly designed to assess ASD symptomatology in individuals with neurogenetic conditions associated with intellectual disability; thus, it is not clear whether ADOS-2 scores (and the results of other assessment tools) should be interpreted in the same way in individuals with neurogenetic conditions compared to so-called idiopathic cases of ASD.

In the present study, we addressed a critical gap in the literature by considering the short-term and long-term psychometric consistency of the ADOS-2 CCS (sometimes also referred to as calibrated severity scores) and classification assignments in individuals with FXS or DS with co-occurring intellectual disability from childhood through young adulthood. Elucidating the extent to which short-term and long-term psychometric consistency is observed in these populations provides critical insights into the assessment and nature of autism symptomatology in these complex phenotypes, as well as clarifies the potential strengths and limitations of the ADOS-2 for use in these conditions.

## Autism Diagnostic Stability in Individuals with ASD

Although ASD is heterogeneous in symptom presentation, and intervention has been found to be effective in supporting more positive developmental outcomes, an autism diagnosis is generally considered to be a stable, lifelong diagnosis (National Collaborating Centre for Mental Health, 2012; Whiteley et al., 2019). Indeed, most individuals with autism will continue to meet clinical thresholds for autism over time despite potential changes in symptom profiles. Across all studies, no information is available regarding the inclusion or stability of autism symptoms in individuals with neurogenetic conditions. For example, Elias and Lord (2022) recently presented data from a longitudinal study of autism symptom trajectories that also considered the long-term stability of ASD diagnoses. The authors presented data from 155 participants for a longitudinal cohort referred for a potential ASD diagnosis for whom assessment data were available at six different time points between 2 and 25 years of age. Overall, approximately 18% of participants in this study had unstable diagnostic impressions (either gained or lost). Moreover, more participants with verbal IQ scores greater than 70 (29% of 69 participants) had unstable diagnostic impressions than those with verbal IQ scores less than 70 (9% of 86 participants). Importantly, in this latter group, changes in diagnostic impressions for half of these participants were not due to changes in ADOS scores, but rather primarily due to changes in clinical decisions regarding whether the participants had intellectual disability only versus intellectual disability and co-occurring ASD.

Other studies have considered potential changes in diagnostic impressions of individuals diagnosed with autism in childhood. For example, Anderson et al. (2014) considered the diagnostic stability at 19 years of age in a sample of 120 children originally diagnosed at 2 years of age. Anderson et al. observed variable diagnostic classifications in approximately 9% of their sample, with more diagnostic changes observed in participants with verbal IQ scores greater than 70 than in those with verbal IQ scores less than 70. Similar rates have been reported in other studies that provided longitudinal follow-ups of individuals with autism diagnosed in childhood (Baghdadli et al., 2018; Billstedt et al., 2005; Howlin et al., 2004; Mawhood et al., 2000).

Finally, a number of studies have considered changes in CCS scores. Recently, Janvier et al. (2022) considered the short-term reliability (short-term assessment intervals ranged from 2 to 8 months) of the ADOS-2 CCS and classification scores in a large sample of individuals with autism (*n* = 608). Overall, results from this investigation demonstrated intraclass coefficients (ICCs) ranging from 0.59 to 0.80 for Modules 1–3, nonsignificant differences in average CCSs, and minimal classification changes. Generally speaking, less than 10% of individuals in the project were observed to change classifications across the short-term assessment period when considering the overall classification and the classifications derived from each domain (i.e., social affective and restricted and repetitive behaviors). Finally, ICC values for the CCSs derived for the overall score and the social affective (SA) domain tended to be higher than those observed for the restricted and repetitive behavior (RRB) domain.

Considering long-term stability, Gotham et al. (2012) used latent class growth curve models in studying a cohort of 345 individuals with best-estimate clinical diagnoses of autism using the ADOS-2 between 2 and 15 years of age. Study authors reported that 258 individuals had 2 or 3 assessment visits and 87 had between 4 and 8 assessment visits; no specific information was provided regarding the time between assessment intervals. Gotham et al. (2012) found that most individuals demonstrated a persistently high or moderate symptom severity level; only 16% received CCSs that changed across time (i.e., improved or worsened). Similarly, Shumway et al. (2012), in an independent sample of 89 children with autism aged 2 to 12 years at the study start and assessed longitudinally across 12 to 24 months, found that 64% of participants earned stable CCS scores (defined as within 1 point); virtually all scores changed by 4 points or less.

## Autism Diagnostic Stability in Individuals with FXS

In addition to being the leading inherited cause of intellectual disability, FXS is also the most common single-gene cause of ASD (Oostra & Willemsen, 2003), with males with FXS often demonstrating a variety of social-affective features associated with ASD along with restricted and repetitive behaviors (e.g., Abbeduto et al., 2014). For example, descriptions of social-affective features include gaze avoidance, limited directed facial expressions, reduced social overtures, and reduced social rapport (Abbeduto et al., 2019; McDuffie et al., 2015; Wolff et al., 2012). The presence of repetitive behaviors, sensory features, and restricted interests are also commonly associated with the FXS phenotype (Abbeduto et al., 2019; McDuffie et al., 2015, Wolff et al., 2012). A few published studies have considered the long-term stability of co-occurring ASD research classifications/diagnoses in individuals with FXS. Across these studies, considerable variability has been observed in findings and in the methodological approaches used to assess autism symptomatology and describe diagnostic stability.

In one study, Hatton et al. (2006) evaluated the stability of ASD classifications using the Childhood Autism Rating Scale (CARS; Schopler et al., 1988) to rate behavioral observations during a one- to two-day assessment visit in a sample of 116 boys (*n* = 99) and girls (*n* = 17) with FXS (mean age = 4.58 years) who had at least two assessment visits averaging 28 months apart. The authors found that approximately 54% of their sample had a stable classification (ASD or no ASD) and 46% had variable ASD classifications.

Hernandez et al. (2009) followed 56 boys with FXS (mean age = 4.72 years, range: 2.75 – 7.33 years) annually for three years and evaluated the stability of a co-occurring ASD diagnosis using a combination of DSM-IV criteria (American Psychiatric, 2000) and the Autism Diagnostic Interview-Revised (ADI-R; Lord et al., 1994). Results indicated a non-significant decrease in the proportion of children diagnosed was observed between the Initial and Longitudinal Follow-Up visits (Initial visit: 42.9%; Retest visit: 43.9%; Longitudinal Follow-Up visit: 35.3%); across all time points, an average agreement rate of 68% was observed. Furthermore, results indicated that of the 24 children who were classified as having ASD (i.e., either pervasive developmental disorder or autism) at the Initial visit, 7 out of the 21 who returned for the Retest visit and 10 out of the 18 who returned at the Longitudinal Follow-Up visit did not retain their diagnosis. In addition, of the 32 children classified as not having ASD at the Initial visit, 4 out of the 24 who returned for the Retest visit and 4 out of the 20 who returned for the Longitudinal Follow-Up visit were reclassified as having ASD.

Finally, Lee et al. (2016) considered the stability of the proportion of individuals classified as ASD in 65 participants with FXS (31 boys and 34 girls) using the ADOS (i.e., the version prior to the ADOS-2) to establish ASD diagnoses across two time points across a mean interval of 2.5 years. Results demonstrated a significant increase in the number of participants earning an autism classification (considering those participants classified as either autism spectrum or autism), with 41.7% of children classified as autistic at the Initial visit (55% of boys and 42% of girls) and 60% of children classified as such at the Retest visit (81% of boys and 41% of girls). Changes in the number of boys with FXS earning ASD classifications largely drove this change. No specific information regarding the number of participants who gained vs. lost ASD classifications were provided by this study.

These studies suggest that a subgroup of participants with FXS demonstrate a variable ASD classification status across time. However, the methodological variability observed across studies makes it difficult to specify the proportion of individuals demonstrating this variable profile and the factors influencing this variability. Moreover, there are no studies that have considered the short-term stability of ASD classifications in individuals with FXS or the stability of their ADOS-2 ASD classifications. Elucidating the extent to which ADOS-2 classification consistency (both short-term and long-term) is observed in this population will provide critical insights into the best approach to assessment and help us begin to characterize the nature of autism symptomatology, as measured by the ADOS-2, in individuals with FXS.

## Autism Diagnostic Stability in Individuals with DS

Although the co-occurrence has been less commonly discussed in the DS literature, there is research to suggest that the prevalence of ASD in individuals with DS falls between 16–42% (DiGuiseppi et al., 2010; Dimachkie Nunnally et al., 2021; Oxelgren et al., 2017; Warner et al., 2017), indicating a prevalence of ASD which, in some cases, falls above that which is seen in the general population (27%; Maenner et al., 2023). Individuals with DS have been reported to exhibit increased difficulties with eye contact, social reciprocity, and spontaneous sharing of interests, as well as increased incidence of stereotyped interests and repetitive behaviors (Capone et al., 2005; Dimachkie Nunnally et al., 2021). Although individuals with DS present with phenotypic challenges in social communication (Channell, 2020; Channell et al., 2015; Fidler, 2005; Hahn et al., 2020; Thurman & Mervis, 2013) and elevated rates of RRBs (Channell et al., 2015, 2019), these rates have been found to be higher in individuals with DS + ASD than those with DS alone (Capone et al., 2005; Dimachkie Nunnally et al., 2021).

To date, only one study has considered the stability of co-occurring ASD in individuals with DS. Hepburn et al. (2008) conducted comprehensive evaluations for 20 toddlers with DS, with a follow-up evaluation conducted for 18 children at four years of age. At two years of age, only three children in this sample were observed to exceed ASD thresholds on the ADOS. All three children continued to be classified in the ASD range on the ADOS at their 4-year-old follow-up evaluation. None of the remaining children were observed to exceed ASD thresholds on the ADOS at their follow-up evaluation. It is important to note that the ADOS classification procedures have changed since the Hepburn et al. study to reflect changes in the DSM criteria used to assess a diagnosis of ASD.

Indeed, the importance of considering the co-occurrence of ASD in individuals with DS has been increasingly recognized (Spinazzi et al., 2023a, 2023b). Only recently have studies begun to consider autism symptomatology more systematically in individuals with DS and the factors influencing variability in their presentation (e.g., Channell et al., 2015; Dimachkie Nunnally et al., 2021; Godfrey et al., 2019; Spinazzi et al., 2023a, 2023b). Elucidating the extent to which the consistency of an ADOS-2 classification (both short-term and long-term) is observed in this population is also needed to provide insight into the best assessment approaches and help us begin to characterize the nature of autism symptomatology, as measured by the ADOS-2, in individuals with DS.

## Present Study

Detailed investigations of autism symptomatology in neurogenetic conditions such as FXS and DS, which are associated with intellectual disability, are critical for understanding the clinical implications of these symptoms and, thus, how best to support individuals. In the present study, we addressed a critical gap in the literature by considering the short-term and long-term psychometric consistency of the ADOS-2 CCSs and classification assignments in individuals with FXS and individuals with DS with an intellectual disability between the ages of 6 and 23 years. Specifically, we examined the short-term consistency of the ADOS-2 scores to provide insight into how stable and reliable the test scores are when administered using an interval that minimizes the influence of external factors and the likelihood of changes in the skills/traits/symptoms themselves. We also consider the long-term consistency of the ADOS-2 scores to provide insight into how stable and reliable the test scores are when administered using an interval more likely to be influenced by changes in development/skills. Applying a standardized context to forming behavioral observations, such as that provided by the ADOS-2, is crucial for framing clinical impressions of an individual’s skills repertoire and can provide valuable insights into our understanding of the nature of autism symptoms in individuals with DS or FXS with intellectual disability.

## Method

### Participants

Participants in the present study were enrolled in a larger project that considered the psychometric properties of samples of expressive language from youth with DS or FXS between 6 and 23 years of age with intellectual disability. Caregivers provided medical reports confirming a diagnosis of DS or FXS (i.e., trisomy 21 or translocation, with or without mosaicism, for youth with DS and the *FMR1* full mutation for youth with FXS). The larger project, from which study data were drawn from, was focused on evaluating the psychometric properties of metrics derived from expressive language samples collected from individuals with intellectual disabilities (ID). This larger project focused on individuals with DS or FXS and co-occurring ID because most individuals with either DS or FXS present with co-occurring ID. Indeed, FXS is the most common inherited cause of ID and DS is the leading genetic cause of ID (Vissers et al., 2016). The following inclusion criteria (based on caregiver report) were utilized in this larger project:

1. Not actively enrolled in a randomized clinical trial in the 8 weeks prior to the testing visits.
2. Use of speech as the primary mode of communication for the youth, with multi-word utterances used at least occasionally.
3. English is the primary language used in the home.
4. No more than a mild hearing loss.
5. No serious (uncorrected) visual impairment that would preclude successful performance on the testing battery.
6. IQ within the ID range (estimated IQ < 75) first determined using caregiver report/record review and subsequently confirmed via direct testing at the initial study visit.

No participants experienced significant changes in physician-prescribed medications designed to manage behavior (e.g., SSRIs), behavioral therapies, or educational programming (not including regular school holidays/vacations) in the eight weeks preceding the testing visits.

Recruitment and testing occurred at four university sites. Of the participants tested, 76 participants with DS (39 males, 37 females), with a mean age of 15.27 years (SD = 5.16, range: 6.45–23.37), and 90 participants with FXS (71 males, 19 females), with a mean age of 14.52 years (SD = 4.68, range: 6.19–23.56), were administered an ADOS-2 at all three study visits (see Table 1 for participant demographics). A comprehensive test battery was administered during the course of a single day for 76% of the sample at the initial study visit; the remaining participants completed the two halves of the initial study visit on different days, with an average of 3.81 days between study half administrations. All participants completed the retest visit in a single day, with a mean test–retest interval of 28.91 days (SD = 5.65, range 18.00–44.00). At the final study visit, which occurred approximately 2 years after the initial visit, assessment measures were administered during the course of a single day for 76% of the sample; the remaining participants completed the two halves of the initial study visit on different days, with an average of 3.81 days between study half administrations. The Institutional Review Boards of all participating universities reviewed and approved study procedures. Written informed consent from the parent/guardian of all participants and youth assent were obtained prior to starting study procedures.

**Table 1:** Participant developmental characteristics at study entry and demographics as a function of diagnostic group

	Fragile X syndrome (n = 90; 71 males, 19 females)				Down syndrome (n = 76; 39 males, 37 females)		
	*M*		*SD*		*M*		*SD*
Chronological Age	14.52		4.68		15.27		5.16
SB-5 Nonverbal Fluid Reasoning	9.74		4.11		10.29		3.17
SB-5 Verbal Knowledge	19.70		6.66		16.32		5.46
SB-5 Abbreviated IQ Change-Sensitive Score	466.84		16.26		462.01		13.39
SB-5 Abbreviated IQ	50.62		6.25		48.50		4.11
Race and Ethnicity	Not Hispanic			Hispanic		Ethnicity Unknown or Prefer not to answer	
	DS	FXS		DS	FXS	DS	FXS
Caucasian	68.4%	77.9%		1.3%	2.2%	0%	0%
Black or African American	4.0%	7.8%		2.6%	0%	0%	0%
Asian	2.6%	1.1%		0%	0%	0%	0%
Other	0%	2.2%		0%	0%	0%	0%
Multi-racial	4.0%	3.3%		1.3%	0%	0%	0%
Race Unknown or Prefer not to answer	0%	0%		15.8%	4.4%	0%	1.1%
Total	79.0%	92.3%		21.0%	6.6%	0%	1.1%

## Measures

The Autism Diagnostic Observation Schedule, Second Edition (Lord et al., 2012) is a semi-structured observational context designed to elicit and observe features central to a diagnosis of ASD (i.e., social-affective skills and the presence of restrictive and repetitive behaviors). One of five ADOS-2 modules is generally selected and administered based on the participant’s developmental and expressive language levels. Because of the level of cognitive delay and the associated limited independence levels of the participants in our sample, none met the criteria for a Module 4 administration. At study entry, 15 participants received Module 1 (4 DS, 11 FXS), 72 participants received Module 2 (37 DS, 33 FXS), and 81 participants received Module 3 (35 DS, 46 FXS). Participants were administered the module most appropriate for their language level; the same module was administered across the short-term interval for 100% of the sample and at all three time points for 85.5% of the sample. Results from the present project focus on considering the stability of ADOS-2 classifications in individuals with DS or FXS. Given the shift in the DSM-5 to one diagnostic category representing “autism spectrum disorder”, individuals classified on the ADOS-2 as having “autism spectrum” or “autism” in the present study were reported as having an ADOS-2 classification as “ASD” and all else classified as “No ASD.”

Overall and domain-level CCSs, which provide an index of symptom severity, were also utilized in study analyses; analyses considering CCSs offer additional insights into the stability of ADOS-2 classifications. The ADOS-2 yields three types of CCS scores: SA CCS, RRB CCS, and Overall CSS, with scores ranging from 0–10. Scores of 4 and above indicate clinically significant autism symptoms.

In addition, there are two methodological issues to note regarding the ADOS-2. First, scores of 4 or above on the SA CSS and RRB CCS alone are not sufficient to earn an ADOS-2 ASD classification. An individual must receive a Total CCS of 4 or above to meet the criteria. In addition, it is important to note that the original RRB CCSs generated by Hus et al. (2014) ranged from 1 to 7, while the SA CCSs ranged from 1 to 10. To address concerns about differing ranges, and to ensure scores of 4 and above indicate clinically significant autism symptomatology, Hus et al. transformed the original RRB scale to a 10-point range, but scores of 2, 3, and 4 are not possible to obtain. Methodological concerns arise within parametric analyses when utilizing a dependent variable in which multiple scores are not obtainable. As such, outside of the descriptive analyses, the original, untransformed RRB CCS is used in study analyses. Second, because the original norming sample did not include participants over 12 years of age for Module 1 and Module 2, the upper age limit for each ADOS-2 module was used to compute the CCSs for any participant older than the ADOS-2 norming sample.

Research-reliable examiners administered the ADOS-2 across all sites. Thirty-five administrations (18 DS and 17 FXS) were randomly selected for cross-site reliability. All examiners at the initial assessment site and the lead site participated in each reliability call. Samples administered by the lead site were evenly distributed for reliability across all other sites. Consensus codes for each reliability administration were achieved through group discussion and the mean percent agreement of each examiner relative to the consensus. Agreement of examiners with the consensus codes averaged 89% across all items for the ADOS-2 (DS: 90%; FXS: 89%); diagnostic agreement averaged 93% (DS: 92%; FXS: 97%).

The Stanford-Binet Intelligence Test, Fifth Edition (Roid, 2003) Abbreviated IQ, which includes the administration of a nonverbal (Fluid Reasoning) and a verbal (Knowledge) subtest, was used to assess cognitive ability. Due to the significant floor effects observed when utilizing the SB-5 with individuals with intellectual disabilities, we utilized the deviation IQ score procedure developed by Sansone et al. (2014), which provides a z-score transformation based on the general population norms. Deviation IQ scores for Abbreviated IQ, Nonverbal Fluid Reasoning raw scores, and Verbal Knowledge raw scores were utilized in the present analyses.

Expressive language ability was assessed by computing metrics of both lexical diversity and mean length of utterance in morphemes during a conversational activity designed to elicit spontaneous speech using procedures developed by Abbeduto and colleagues (Abbeduto et al., 2020, 2023; Thurman et al., 2021). In this interview-style conversation, the examiner’s behavior was scripted to ensure consistency across administrations and limit the amount of examiner talk and the extent to which the examiner prompted or scaffolded the participant’s talk. Samples are audio-recorded in quiet testing rooms, with the examiner and participant seated together at a table for a goal of 12 min. Participant speech was transcribed, segmented into C-units (communication units, which could range from a single-word utterances to an independent clause and its modifiers), and analyzed using the computer algorithm for computing mean length of utterance in morphemes (MLU-m) in the software program Systematic Analysis of Language Transcripts (SALT; Miller & Iglesias, 2008). Details of the conversational scripts, examiner prompts, transcription, segmentation, and computation of MLU-m, as well as procedures for evaluating inter-transcriber agreement, can be found in Abbeduto et al. (2020) and Thurman et al. (2021). These metrics were utilized in follow-up analyses to consider the potential influences of participant characteristics on stability of ASD classifications.

## Analysis Plan

The present study analyses focus on considering the short-term and long-term consistency of the ADOS-2 CCSs and ADOS-2 classification assignments in individuals with FXS or DS from childhood through young adulthood. Due to non-normal distributions, nonparametric analyses were used to compare the participants with DS and participants with FXS on chronological age, SB-5 Nonverbal Fluid Reasoning raw score, SB-5 Verbal Knowledge raw scores and ADOS-2 CCSs. We corrected for multiple comparisons by using Benjamini and Hochberg’s false discovery rate (Benjamini & Hochberg, 1995). Next, we considered the short-term and long-term consistency of the ADOS-2 CCSs. Specifically, Friedman’s ANOVAs were conducted to evaluate whether CCSs changed over time and two-way random absolute Intraclass Correlation Coefficients (ICCs) for test–retest reliability were calculated for Overall CCSs, SA CCSs, and the RRB CCSs. In addition, we used descriptive statistics to consider both the short-term and long-term consistency of ADOS-2 classifications (ASD vs No ASD). Finally, exploratory follow-up analyses were conducted to gain insight into the participant factors influencing stable vs. unstable ASD classifications in each group. Further, we compared CCS change scores across the FXS and DS groups and, for each group, we considered the potential influences of selected participant characteristics (e.g., ADOS-2 CCS, nonverbal ability, and verbal ability) on whether participants were stable vs. unstable in their ADOS-2 classifications.

## Results

### Descriptive Analyses

Table 1 displays the developmental characteristics of each participant group at study entry. No significant differences were observed between the participants with DS and participants with FXS on chronological age (*U* = 3116.50, *p* = 0.33, *r* = 0.08) or SB-5 Nonverbal Fluid Reasoning raw score (*U* = 3541.50, *p* = 0.18, *r* = 0.11). A significant difference was observed between the two groups on SB-5 Verbal Knowledge raw scores (*U* = 4313.00, *p* < 0.001, *r* = 0.29), with participants with DS, as would be expected, earning significantly lower verbal raw scores than participants with FXS. Analyses were also conducted to compare the ADOS-2 CCS scores (i.e., overall, SA domain, and RRB domain at the initial test visit). Results from these analyses demonstrated that participants with DS earned significantly lower ADOS-2 CCS overall scores (*U* = 5263.50, *p* < 0.001, *r* = 0.47), in the SA domain (*U* = 5061.50, *p* < 0.001, *r* = 0.42), and in the RRB domain (*U* = 4936, *p* < 0.001, *r* = 0.39). All significant findings remained after FDR correction. At the initial assessment visit, 37.2% of participants with DS and 75.6% of participants with FXS earned an autism classification on the ADOS-2.

## Consistency: ADOS-2 Calibrated Severity Scores

**Fig. 1:**
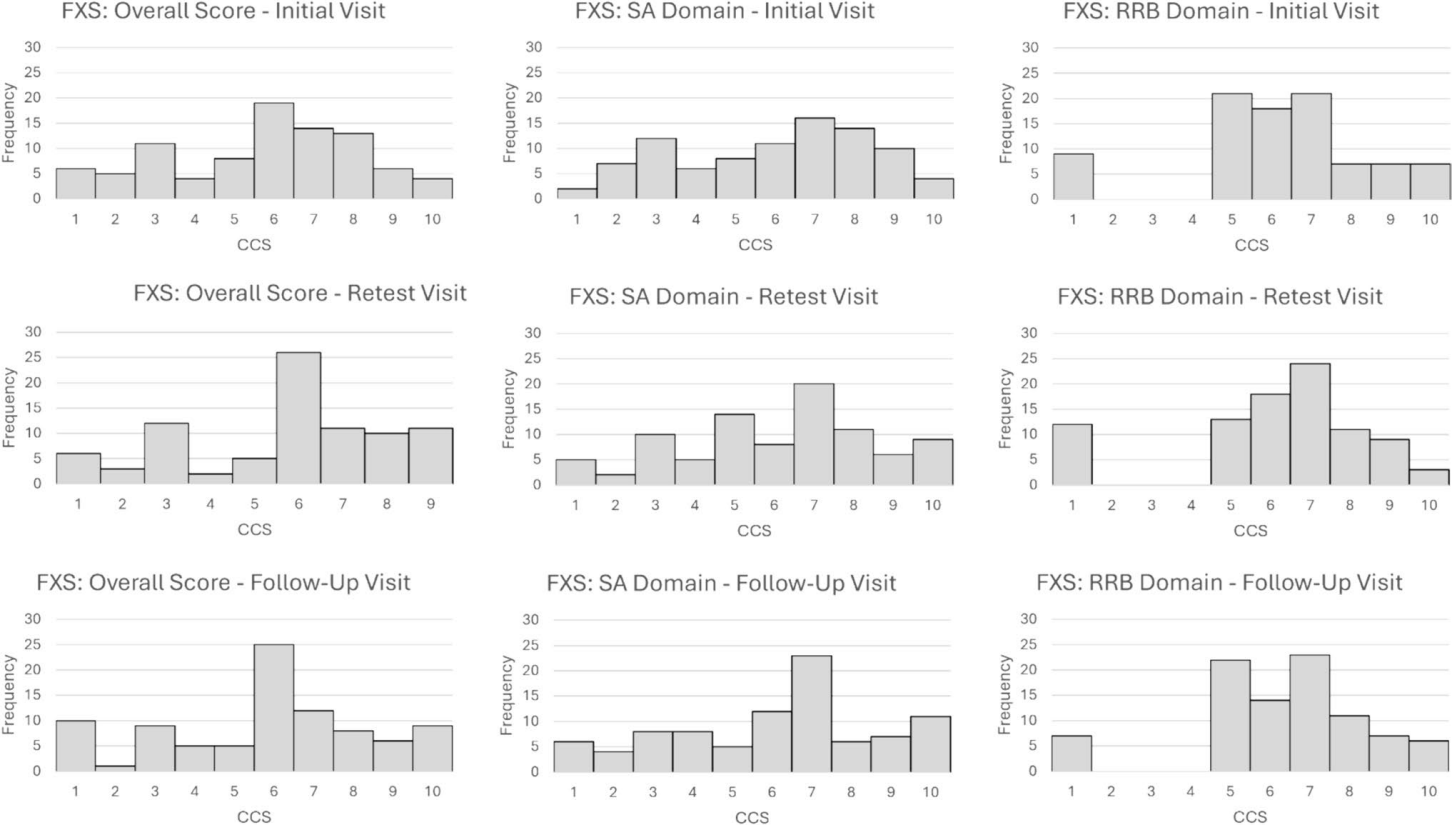
Histograms of ADOS-2 Overall, Social Affect, and Restricted and Repetitive Behavior Calibrated Comparison Scores for participants with fragile X syndrome

**Fig. 2:**
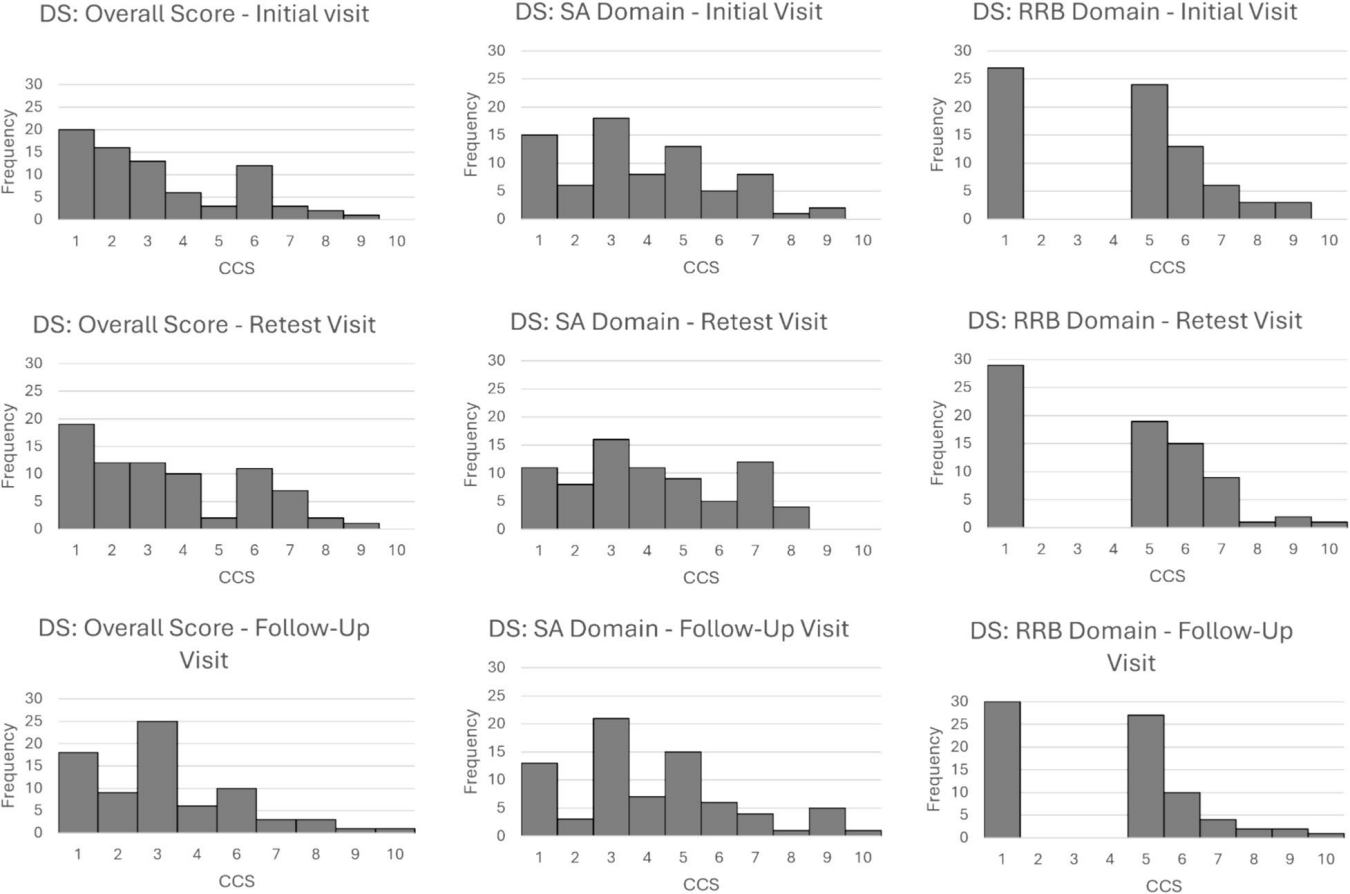
Histograms of ADOS-2 Overall, Social Affect, and Restricted and Repetitive Behavior Calibrated Comparison Scores for participants with Down syndrome

Friedman’s ANOVAs were conducted to assess whether there were differences across the assessment visits in the CCS scores (see Table 2). For each diagnostic group, no significant differences were observed across the different assessment visits for the Overall CCS (DS: χ^2^(2) = 2.35, *p* = 0.31; FXS: χ^2^(2) = 0.24, *p* = 0.89), SA CCS (DS: χ^2^(2) = 0.24, *p* = 0.89; FXS: χ^2^(2) = 1.51, *p* = 0.47), or RRB CCS (DS: χ^2^(2) = 0.78, *p* = 0.68; FXS: χ^2^(2) = 0.72, p = 0.70). Histograms of the Overall, SA, and RRB CCS scores for both participants with DS and participants with FXS are provided in Figs. 1 and 2, respectively.

**Table 2:** ADOS-2 Calibrated Comparison Scores: Short-term and long-term administration visits

	Initial visit		Retest visit		Follow-up visit	
	*M*	*SD (range)*	*M*	*SD (range)*	*M*	*SD (range)*
	Fragile X syndrome (n = 90)					
Overall-CCS	5.72	2.44 (1–10)	5.92	2.45 (1–10)	5.84	2.62 (1–10)
SA-CCS	5.96	2.44 (1–10)	6.10	2.47 (1–10)	6.10	2.61 (1–10)
RRB-CCS	6.50	1.70 (4–10)	6.53	1.62 (4–10)	6.60	1.65 (4–10)
	Down syndrome (n = 76)					
Overall-CCS	3.26	2.16 (1–9)	3.54	2.27 (1–9)	3.41	2.23 (1–10)
SA-CCS	3.82	2.15 (1–9)	4.08	2.15 (1–8)	4.11	2.15 (1–10)
RRB-CCS	5.25	1.37 (4–9)	5.26	1.37 (4–10)	5.09	1.32 (4–10)
Notes

We also computed the ICCs between the ADOS-2 CCSs across all three of the assessment visits (see Table 3). For each diagnostic group, ICCs were significant and remained significant after FDR correction. Moreover, for both participants with DS and participants with FXS, the ICCs for RRB CCSs were generally lower than for the SA and Overall CCSs, with the ICCs for participants with DS dipping into the poor range. For participants with FXS, with the exception of the Initial vs. Longitudinal Follow-Up visit ICC for the RRB CCSs, moderate to good ICCs were observed across all visits and CCSs. ICCs for the participants with DS were a bit lower than those observed for the participant with FXS, with their Initial-Retest visit ICCs comparisons also dipping into the poor range.

**Table 3:** ICCs for the ADOS-2 Calibrated Comparison Scores as a function of diagnostic group

	Initial vs. Retest Visit Comparison ICC (95% CI)	Initial vs. Follow-up visit Comparison ICC (95% CI)	Retest vs. Follow-up visit Comparison ICC (95% CI)
	Fragile X syndrome (n = 90)		
Overall-CCS	.809(.723–.870)^**^	.545 (.382–.676)^**^	.649 (.511–.754)**
SA-CCS	.775(.678–.846)^**^	.569(.411–.694)^**^	.606 (.457–.722)**
RRB-CCS	.646 (.507–.752)^**^	.480 (.303–.624)^**^	.528 (.361–.663)**
	Down syndrome (n = 76)		
Overall-CCS	.700 (.566–.799)^**^	.417 (.212–.587)^**^	.569 (.394–.703)**
SA-CCS	.656 (.508–.767)^**^	.483(.291–.637)^**^	.549 (.370–.689)**
RRB-CCS	.432 (.229–.599)^**^	.252 (.029–.451)^*^	.193 (− .032–.400)*
*p < .05, **p < .001			

## Consistency: ADOS-2 Classification Scores

We also considered the short-term consistency of ADOS-2 classifications (ASD vs No ASD) for the participants with DS and the participants with FXS (see Table 4). Results indicated that significantly more participants with DS than FXS had overall classifications that differed across the short-term interval (*U* = 4968.00*, z* = 5.11, *p* < 0.001, *r* = 0.39). From the perspective of the stability of an ASD classification, of the participants classified in the ASD concern range at either the test or retest visits (DS: *n* = 40; FXS: *n* = 74), 40% with DS and 15% of participants with FXS demonstrated a variable classification. Moreover, of the participants who demonstrated variable classifications, 31.4% of participants with DS and 45.5% of participants with FXS gained a classification at the re-test visit, whereas 68.6% of participants with DS and 54.5% of participants with FXS lost the classification at the re-test visit. Moreover, 75% of our participants with DS who received variable classifications and 77% of those with stable classifications were assessed by different examiners across the two administrations. Similarly, among participants with FXS, 54.5% of those with variable classifications and 71.4% of those with stable classifications were evaluated by different examiners during the two sessions.

**Table 4:** Short-Term Consistency in ADOS-2 classification in participants with FXS or DS

	Not classified	Variable Classification	Classified
FXS (*n* = 90)			
Overall	17.8%	12.2%	70.8%
SA Domain	15.6%	11.1%	73.3%
RRB Domain	5.6%	12.2%	82.2%
Down syndrome (*n* = 76)			
Overall	50.0%	21.1%	28.9%
SA Domain	35.5%	26.3%	38.2%
RRB Domain	20.5%	30.8%	48.7%

**Fig. 3:**
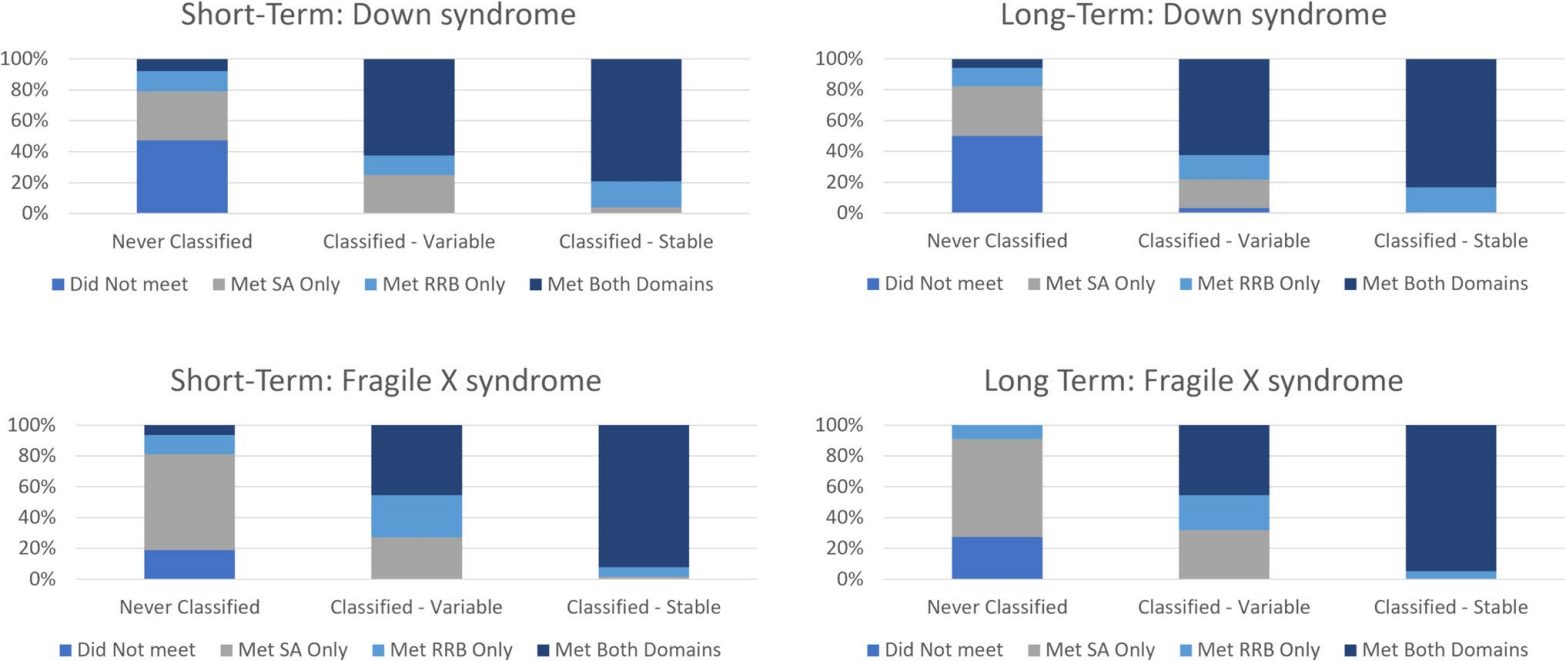
ADOS-2 domain patterns at study entry (initial visit) as a function of diagnostic group and short-term vs. long-term stability period. Figures along the left-side panel represent those “never classified”, “classified-variable”, and “classified-stable” across the short-term stability period (i.e., initial-retest visits). Figures along the right-side panel represent those “never classified”, “classified-variable”, and “classified-stable” across the entire project period (i.e., initial, retest, and longitudinal follow-up visits)

A similar pattern of findings was observed when considering short-term classification variability for both the SA and RRB domains. For example, of the participants classified in the ASD concern range at either the test or retest visits on the SA domain (DS: *n* = 51; FXS: *n* = 76), variable classifications were observed for 39.2% of participants with DS vs. 13.2% of participants with FXS (proportion of variable group gained a classification at re-test visit: DS = 40.0%, FXS = 30.0%,). Of the participants classified in the ASD concern range at either the test or retest visits on the RRB domain (DS: *n* = 62; FXS: *n* = 85), variable classifications were observed for 38.7% of participants with DS vs. 12.9% of participants with FXS (Proportion of variable group gained at re-test visit: DS = 54.2%, FXS = 63.6%). As seen in Fig. 3, in each diagnostic group, variability was observed in the ADOS-2 domain outcomes (e.g., did not meet either domain, SA only, RRB only, met on both domains) at the initial visit, regardless of stability pattern (e.g., those who were never classified, those with variable classifications, and those with stable classifications).

The consistency of classification scores for individual participants was also considered across all three timepoints (i.e., initial visit, retest visit, and longitudinal follow-up visit; see Table 5 for summary of classifications across time points). For each diagnostic group, the percentage of participants with variable ADOS-2 classifications increased relative to the numbers observed when comparing the test–retest visit sessions. For example, of the participants classified in the ASD concern range at any point in the project (DS: *n* = 44; FXS: *n* = 79), 71.4% with DS and 27.8% of participants with FXS demonstrated a variable classification; of these participants 87.5% of participants with DS and 90.9% of participants with FXS received the same ADOS-2 module across all time points. Similar to the overall classification score findings, of the participants classified in the ASD concern range on the SA domain at any point in the project (DS: *n* = 57; FXS: *n* = 80), 57.9% with DS and 25% of participants with FXS demonstrated inconsistent ASD classifications. On the RRB domain, of the participants classified in the ASD concern range (DS: *n* = 68; FXS: *n* = 89), 87.2% with DS and 20.2% of participants with FXS demonstrated inconsistent ASD classifications in the RRB domain. As seen in Table 6, of the participants who were observed to demonstrate classification inconsistency across the project, half demonstrated short-term inconsistency and half demonstrated short-term stability. In addition, in each diagnostic group, variability was observed in the number of domains on which individuals exceeded threshold scores regardless of their classification status (see Fig. 3).

**Table 5:** Consistency in ADOS-2 Classification over a 2-year interval: CCS score 4 or higher

	Never classified	Classified at 1 visit	Classified at 2 visits	Consistently Classified
Fragile X syndrome (n = 90)				
Overall	12.2%	8.9%	15.6%	63.3%
SA Domain	11.1%	6.7%	15.6%	66.7%
RRB Domain	1.1%	7.8%	12.2%	78.9%
Down syndrome (n = 76)				
Overall	43.6%	15.4%	25.6%	15.4%
SA Domain	26.9%	20.5%	21.8%	30.8%
RRB Domain	12.8%	17.9%	35.9%	33.3%

**Table 6:** ADOS-2 classification patterns across the 2-year project period

Classification Profile Across Visits			Diagnostic Group	
Met at Initial Visit?	Met at Retest Visit?	Met at Follow-Up Visit?	DS (%)	FXS (%)
No	No	No	44.7	12.2
No	No	Yes	5.3	5.6
No	Yes	No	5.3	0
No	Yes	Yes	9.2	6.7
Yes	No	No	5.3	3.3
Yes	No	Yes	1.3	2.2
Yes	Yes	No	13.2	6.7
Yes	Yes	Yes	15.8	63.3

## Follow-Up Analyses: Features Influencing ASD Classification Variability

**Fig. 4:**
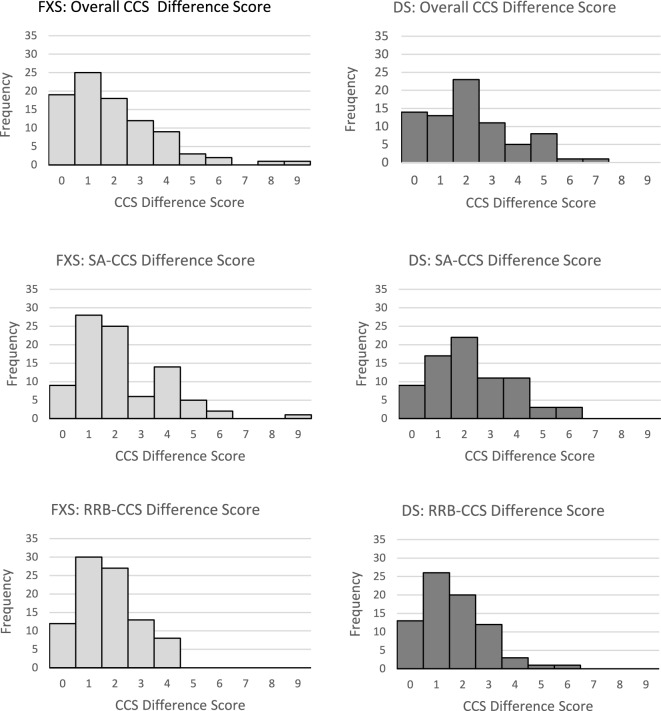
Distributions of maximum ADOS-2 CCS difference score (i.e., largest CCS score earned minus the smallest CCS score earned across the three administrations) as a function of diagnostic group

Follow-up analyses were then conducted to gain insight into the potential characteristics contributing to ASD classification variability; all significant effects reported remained significant after applying FDR correction. To begin, we considered the presence of differences in the ADOS-2 project lifetime CCSs (i.e., project lifetime maximum CCS minus minimum CCS; see Fig. 4 for distributions of scores) between youth with DS and youth with FXS; that is, the highest CCS score earned across the project (i.e., initial, retest, or longitudinal follow-up visit), minus the smallest CCS score earned across the project (i.e., initial, retest, or longitudinal follow-up visit). No significant between-group differences were observed in the overall (*U* = 3087.00, p = 0.27, *r* = 0.09), SA domain (*U* = 3262.00, *p* = 0.60, *r* = 0.04), or RRB domain CCSs (*U* = 3606.00, *p* = 0.53, *r* = 0.05).

Next, within each diagnostic group, we considered whether participants with stable vs. variable ASD classifications varied from one another in terms of overall CCSs at study entry, CA, language scores, and cognitive ability (see Table 7 for descriptive statistics). In each diagnostic group, participants with variable ASD classifications earned significantly lower overall CCS scores at study entry (DS: *U* = 45.00, *p* < 0.001, *r* = 0.59; FXS: *U* = 132.00, *p* < 0.001,* r* = 0.62) and had significantly higher lexical diversity (DS: *U* = 298.00, *p* = 0.001, *r* = 0.51; FXS: *U* = 968.50, *p* < 0.001, *r* = 0.42), and syntactic complexity (DS: *U* = 304.00, *p* < 0.001, *r* = 0.53; FXS: *U* = 971.50, *p* < 0.001, *r* = 0.42) than did participants with stable ASD classifications. For participants with FXS, but not those with DS, cognitive ability scores at study entry were also lower for participants with stable ASD classifications than for those with variable ASD classifications (FXS: *U* = 900.00, *p* < 0.001, *r* = 0.39; DS: *U* = 211.50, *p* = 0.05, *r* = 0.01).

**Table 7:** Descriptive statistics for follow-up analyses as a function of diagnostic group

	Stable ASD Classification (FXS: *n* = 57; DS: *n* = 12)		Variable ASD Classification (FXS: *n* = 22; DS: *n* = 30)		Stable No ASD Classification (FXS: *n* = 11; DS: *n* = 34)	
	*M*	*SD*	*M*	*SD*	*M*	*SD*
	Fragile X syndrome					
Chronological Age	14.17	4.91	14.28	4.40	16.78	3.60
SB-5 Abbreviated IQ Change-Sensitive Score	460.79	14.81	475.24	14.80	482.18	6.79
ADOS-2 Overall CCS	7.11	1.36	3.95	2.13	2.09	0.94
Lexical Diversity	59.51	29.89	89.45	28.29	100.55	36.12
Syntactic Complexity	2.88	1.21	4.07	1.18	4.80	1.76
	Down syndrome (12 vs. 30 vs. 34)					
Chronological Age	15.17	5.70	16.13	4.86	14.57	5.37
SB-5 Abbreviated IQ Change-Sensitive Score	455.60	6.90	460.75	13.41	463.87	15.27
ADOS-2 Overall CCS	6.50	1.31	3.75	1.88	1.76	0.78
Lexical Diversity	39.50	22.81	69.59	30.78	85.53	34.67
Syntactic Complexity	1.98	0.78	3.15	1.31	3.95	1.70

Within each diagnostic group, when participants with variable ASD classifications were compared to those consistently classified as not ASD, participants with variable ASD classifications earned significantly higher overall CCS scores at study entry (DS: *U* = 45.00, *p* < 0.001, *r* = 0.53; FXS: *U* = 132.00, *p* < 0.001, *r* = 0.48). For participants with FXS, but not DS, cognitive ability scores at study entry were also lower for participants with variable ASD classifications (FXS: *U* = 167.00, *p* = 0.04, *r* = 0.36; DS: *U* = 522.00, *p* = 0.55, *r* = 0.08) than those consistently classified as not ASD. For each diagnostic group, there were no significant differences between participants with variable ASD classifications and those consistently classified as not ASD on lexical diversity (DS: *U* = 626.00, *p* = 0.12, *r* = 0.20; FXS: *U* = 139.50, *p* = 0.49, *r* = 0.12) or syntactic complexity (DS: *U* = 626.00, *p* = 0.12, *r* = 0.20; FXS: *U* = 140.50, *p* = 0.46, *r* = 0.13).

## Discussion

DS and FXS are each associated with a higher occurrence of autism symptomatology relative to the general population (Clifford et al., 2007; DiGuiseppi et al., 2010; Dimachkie Nunnally et al., 2021; Harris et al., 2008; Kaufmann et al., 2017; Klusek et al., 2014; Oxelgren et al., 2017; Warner et al., 2017). The assessment of autism in both populations is complex due to the presence of other co-occurring behavioral features that can influence the presentation of autism symptomatology, such as intellectual disability and language delays. In the present study, we fill a critical gap in the literature by reporting on the short-term and long-term consistency of the ADOS-2 CCSs and classification assignments in individuals with DS or FXS from childhood through young adulthood. Such data can be valuable for understanding the nature of autism symptomatology in these complex phenotypes and clarifying the potential strengths and limitations of the ADOS-2 for use with individuals with these conditions.

When examining the consistency of the ADOS-2 CCS scores in the two diagnostic groups, there were no significant differences across assessment visits (i.e., short-term and long-term) on any of the CCSs. ICCs were also significant across all comparisons. Specifically, short-term reliability scores were relatively similar to findings reported by Janvier et al. (2022) regarding the test–retest reliability of the ADOS-2 CCS scores in individuals with idiopathic autism, with ICC values for the CCSs derived for the overall score and the SA domain generally higher than those observed for the RRB domain. That said, the reliability of the RRB CCS score in individuals with DS was lower than that observed for our participants with FXS as well as the values reported by Janvier et al. (2022) for their participants with autism. Moreover, in both diagnostic groups, reliability scores across all three time points were lower than those observed in the short-term (test–retest) period. Similar to the findings for the short-term domain score reliability comparisons, reliability scores across all three timepoints for the CCSs derived for the overall score and the SA domain were generally higher than those observed for the RRB domain. The long-term reliability scores for the RRB CCS in individuals with DS also did not meet statistical significance.

Findings relating to the decreased reliability of the RRB CCS in individuals with DS relative to those with FXS and, for both diagnostic groups, relative to the other CCS scores are likely due to the measurement challenges associated with the assessment of RRB and between-group differences related to the phenomenology of RRBs. It is widely acknowledged that RRBs are challenging to assess directly, particularly during short observational periods, because they may only occur during particular conditions or intermittently. Indeed, Lord et al. (Hus et al., 2014; Lord et al., 2006) note that “while the presence of RRBs during this brief observation may be clinically significant, the absence of these behaviors in this time-limited, standardized context must be interpreted more cautiously (Hus et al., 2014, p. 2401)”. Thus, the measurement of RRBs in individuals who present with a milder presentation of RRB or a different phenomenological profile due to the presence of cognitive delays may require additional sources of information when considering the co-occurrence of autism (Channell et al., 2015; Evans et al., 2014; Glenn & Cunningham, 2007; Hepburn & MacLean, 2009; Moskowitz et al., 2020; Oakes et al., 2016; Reisinger et al., 2020; Stores et al., 1998). Moreover, investigations focused on characterizing the presence and phenomenological profiles of RRBs in individuals with complex clinical phenotypes, such as DS and FXS, would likely increase our understanding regarding the mechanisms underlying the presence of RRBs in this population, which could, in turn, increase clinical confidence regarding when to consider the co-occurrence of an autism diagnosis.

Although no significant differences were observed for the ADOS-2 CCSs across short-term and long-term comparisons for our participants with DS or FXS, comparisons of the ADOS-2 classification assignments provide a different perspective regarding the stability of autism symptomatology. When considering the short-term and long-term classification stability, significantly more participants with DS than those with FXS demonstrated variable classifications. For both groups, the proportion of participants who were classified in the ASD concern range at any point in the project (at any of the three timepoints) was greater than the proportion classified at the initial visit, with approximately half of participants with DS and the vast majority of participants with FXS earning a classification in the ASD range at some point during the project period. Although it is widely accepted that individuals with DS or FXS are at increased risk of autism relative to the general population, youth are rarely evaluated for the co-occurring presence of autism (Klusek et al., 2023; Spinazzi et al., 2023a, 2023b). Thus, investigations clarifying the nature of autism symptomatology in these populations and supporting improvements in the assessment and diagnosis of co-occurring ASD are vital for ensuring these families receive necessary treatment and educational supports. Our findings suggest that the identification and diagnosis of autism in these neurogenetic conditions is vital for ensuring access to appropriate interventional and educational support in light of the high rates of co-occurrence of autism. In addition, there is also evidence that suggests that individuals with DS or FXS with co-occurring autism may also be at increased risk of a variety of medical conditions (García‐Nonell et al., 2008; Kaufmann et al., 2017; Spinazzi et al., 2023a, 2023b); thus, improving the identification and diagnosis of ASD in these populations may facilitate medical management in these populations. We also gained additional insights into the assessment of ASD in these populations by considering the data from the participants earning classifications in the ASD range. Specifically, we observed a subgroup of participants with FXS who earned variable short-term classifications; this number nearly doubled when considering the proportion of participants who demonstrated variable long-term classifications. For participants with DS, almost half demonstrated variable short-term ASD classifications; this number increased with nearly three-quarters of the sample earning variable long-term ASD classifications. For each diagnostic group, we observed similar patterns of findings for both the SA and RRB domains. Most youth earning classifications in the ASD range received the same ADOS-2 module across all time points and changes, suggesting that module changes do not contribute to classification variability. There was a trend, more so for participants with DS, across the short-term period to be less likely to gain than lose their classification; across the long-term period for both diagnostic groups, patterns of change were pretty evenly distributed, with half of those with unstable classifications demonstrating short-term stability and half demonstrating short-term instability.

Finally, follow-up analyses provide insight into the potential factors influencing classification instability. First, across the 2-year project period, no between-group differences were observed in overall or domain CCS change scores (i.e., project maximum CCS minus project minimum CCS). These data highlight the fact that the increased rate of classification instability is not due to between-group differences in CCS scores across assessments. Second, for both diagnostic groups, participants with variable ASD classifications tended to earn ADOS-2 CCSs and language scores at study entry that fell between participants who were never classified as ASD and participants who were consistently classified as ASD. For participants with FXS, those with variable ASD classifications also earned higher cognitive ability scores that fell between those who never obtained an ASD classification and those with stable ASD classifications. Together, these data suggest that our finding that significantly more participants with DS than those with FXS demonstrate variable classifications may have to do with where individuals with DS fall on the continuum of autism features relative to participants with FXS. That is, the increased likelihood of observing variable autism classifications in the participants with DS may be because, as a group, more participants with DS earned scores in the sub-clinical/mild symptom presentational range, and therefore, fluctuations in CCS scores are more likely to impact classifications than would be observed in groups who demonstrate higher CCS scores, like the participants with FXS.

Indeed, autism symptom presentation is heterogeneous across participants and age within individuals (Gotham et al., 2007, 2012). Nonetheless, most individuals with autism will continue to meet clinical thresholds for autism over time despite potential changes in symptom profiles (Anderson et al., 2014; Baghdadli et al., 2018; Billstedt et al., 2005; Elias & Lord, 2022; Howlin et al., 2004; Mawhood et al., 2000). That said, we must consider how phenotypic presentations can influence the evaluation of autism symptomatology in different populations. For example, the presentation of autism symptomatology in individuals with DS or FXS is known to be milder on average relative to individuals with autism without co-occurring neurogenic conditions (e.g., Dimachkie Nunnally et al., 2021; Thurman & Hoyos Alvarez, 2020; Thurman et al., 2015). This factor alone makes the evaluation of autism in these populations more clinically complex, given that milder presentations of ASD are more difficult to evaluate (e.g., Gibbs et al., 2012; Mayes et al., 2013; McPartland et al., 2012).

Moreover, because autism symptoms vary across development, it is not clear how such symptoms will present in individuals with co-occurring intellectual disability or how variable cognitive and behavioral phenotypes will influence the presentation of autism symptomatology over time. Nonetheless, many individuals with DS or FXS demonstrate autism symptomatology significant enough to warrant clinical monitoring and evaluation. In addition, more research focused on unpacking the full range of heterogeneity of autism symptomatology and the factors influencing this heterogeneity in individuals with DS and FXS is needed to facilitate the assessment of autism symptomatology in these clinically complex conditions. Such data would likely facilitate the diagnosis and assessment of autism in these populations as many providers may not be confident discerning autism symptomatology from the phenotypic and developmental presentations of these conditions.

### Limitations and Future Directions

Although the present study begins to fill a critical gap in the literature regarding the assessment of autism in individuals with DS or FXS, limitations should be noted. First, although the sample size of the present study is large compared to most of the literature in the field using direct assessment methods, studies using larger sample sizes or more specific ages/developmental periods are needed to describe the nature and quality of symptom expression more precisely. For example, because the ADOS-2 considers the severity of autism symptomatology relative to language and age, additional research is required to unpack the extent to which findings of stability vs. variability are apparent at different ages and developmental levels. In addition, because of the cognitive delays associated with many neurogenetic conditions, affected individuals required the administration of ADOS-2 modules (e.g., single words and phrase-level speech) that were not normed for their ages. Future studies considering the symptom presentation using new tools specifically designed for these populations, such as the Adapted-ADOS (Bal et al., 2020), are needed to understand symptom presentation fully. Additionally, large-scale studies on the measurement invariance of the ADOS-2 would enhance our understanding of how this tool functions in individuals with neurogenetic conditions. Finally, for a more comprehensive understanding of symptom stability over time, large-scale studies are needed utilizing tools designed specifically to monitor changes in symptom presentation, such as the Brief Observation of Social Communication Change (BOSCC).

The present study also is limited by its sample characteristics. Specifically, eligibility for the larger study from which data were drawn required that participants occasionally produce 3-word utterances based on caregiver report and have an IQ score less than 75. Although these eligibility criteria allowed for the inclusion of most individuals with DS or FXS in this age range, more research is needed to consider the ADOS-2 performance of individuals with more limited language ability and those with more advanced cognitive ability. Indeed, it is vital that future studies be as inclusive as possible when considering autism symptomatology in these populations to ensure the full range of diversity in skills represented. In addition, few females with FXS were included in the present study. The prevalence of FXS is more common in males than in females due to the X-linked nature of the condition; moreover, females on average are less affected than their male counterpart and thus are less likely than their male counterparts to meet the study criteria requiring participants to demonstrate an IQ score less than 75 (Hunter et al., 2014). Thus, research specifically focused on understanding autism symptom presentation in females with FXS are much needed. Additionally, the demographics of the current sample lacks representation across minoritized groups. Not only should future studies include samples with more diverse racial and ethnic identities, but should also consider racial, ethnic, and sociodemographic disparities in the diagnosis and assessment of co-occurring ASD in individuals with DS or FXS.

In addition, due to the study design, we were only able to conduct exploratory analyses considering some potential factors contributing to ADOS-2 classification variability, focusing on language and overall cognitive ability. Detailed investigations considering agreement and use of multiple assessment methods, stability at a symptom level, and other behavioral and medical features that potentially could influence the presentation of autism symptomatology (e.g., ADHD, anxiety, sensory difficulties, co-occurring medical conditions) are needed to support clinical confidence in the assessment of autism in these phenotypically complex populations. There are multiple avenues to consider for future research to expand our understanding in this area. For example, future studies may also consider the inclusion of an idiopathic autism comparison group (i.e., autism only) or consider detailed investigations of autism symptoms across development in neurogenetic conditions to shed further light on the presentation of ASD in neurogenetic conditions, and how this presentation may change over time. With these data, it is likely that specific assessment guidelines and parameters can be formed to facilitate the screening and diagnosis of autism in these populations.

Finally, clinical best estimate diagnoses were not available for the participants in the present study, which limits our ability to explore the relationship between variability in ADOS-2 classification and stability of clinical diagnosis. Indeed, studies that consider both the presence of autism symptomatology across all individuals with neurogenetic conditions, such as DS or FXS, and clinical best estimate diagnoses are needed to elucidate whether the ADOS-2 consistently classifies participants that have a clinical diagnosis of autism. At the same time, it is vital that investigations of the co-occurring presence of autism in individuals with neurogenetic conditions continue to reflect on and question what the assessment tools utilized in these evaluation procedures are and are not capturing to ensure we continue to advance both research and clinical practices.

## Conclusions

Although the likelihood of presenting with autism symptomatology is higher for individuals with various neurogenetic conditions, especially those associated with intellectual disability such as DS and FXS, ASD is often diagnosed later or not at all in these populations (Howlin et al., 2008; Klusek et al., 2014; Spinazzi et al., 2023a, 2023b). As such, affected individuals may miss intervention opportunities that have the potential to improve developmental outcomes. Research focused on improving our understanding and ability to assess autism symptomatology in these clinically complex populations is vital for education and for improving our ability to screen and diagnose ASD in these populations. The present study fills a critical gap in the literature by considering the short-term and long-term consistency of the ADOS-2 CCSs and classification assignments in individuals with FXS or DS from childhood through young adulthood. Results from the present study highlight not only the high frequency of autism symptomatology in individuals with DS or FXS but also the fact that many individuals can fall into a gray area in their symptom expression, with variability across both short-term and long-term periods when presented with structured observational tasks such as the ADOS-2. Future studies considering the heterogeneity of autism symptomatology and developmental influences on the form and quality of symptoms are needed to help guide providers in the assessment and diagnosis of co-occurring autism in these populations. Moreover, results from the present study highlight the potential influence of cognitive and linguistic abilities on ADOS-2 classification stability. It is likely that these factors, as well as others such as co-occurring behavioral features (e.g., anxiety, inattention, hyper-activity), influence measurement stability and clinical observations. Indeed, across development, expectations for success, and thus the skills needed to adapt to these situations, change. Thus, investigations across the lifespan are crucial for understanding autism symptomatology in these populations.
